# EGR2 is a hub-gene in myocardial infarction and aggravates inflammation and apoptosis in hypoxia-induced cardiomyocytes

**DOI:** 10.1186/s12872-022-02814-3

**Published:** 2022-08-15

**Authors:** Zhixiang Bo, Shuwen Huang, Li Li, Lin Chen, Ping Chen, Xiaoyi Luo, Fang Shi, Bing Zhu, Lin Shen

**Affiliations:** 1 Department of Thoracic and Cardiovascular Surgery, The Second Affiliated Hospital of Chongqing Medical University, #76 Linjiang Road, Yuzhong District, Chongqing, 400010 China; 2 Research Base of Traditional Chinese Medicine Syndrome, Fujian University of Traditional Chinese Medicine, Fuzhou, 350122 China; 3 Department of Surgery, Wushan County Hospital of Traditional Chinese Medicine, Chongqing, 400010 China; 4 Department of Gastroenterology, The Fifth People’s Hospital of Chongqing, Chongqing, 400010 China

**Keywords:** Myocardial infarction (MI), Cardiomyocytes, Hub-gene, Hypoxia, EGR2

## Abstract

**Background:**

Myocardial infarction (MI) is characterized by coronary artery occlusion, ischemia and hypoxia of myocardial cells, leading to irreversible myocardial damage. Therefore, it is urgent to explore the potential mechanism of myocardial injury during the MI process to develop effective therapies for myocardial cell rescue.

**Methods:**

We downloaded the GSE71906 dataset from GEO DataSets, and the R software was used to identify the differentially expressed genes (DEGs) in mouse heart tissues of MI and sham controls. Gene Ontology (GO) and Kyoto Encyclopedia of Genes and Genomes (KEGG) pathway enrichment were performed to understand the significantly activated signaling pathways in MI. Protein–protein interaction (PPI) network was constructed to highlight the hub genes in DEGs. The Western Blot, qRT-PCR and TUNEL staining were used to explore the function of hub gene in hypoxia-induced cardiomyocytes in vitro.

**Results:**

A total of 235 DEGs were identified in GSE71906 dataset. Functional enrichment analysis revealed that the upregulated genes were primarily associated with the inflammatory response and apoptosis. 20 hub genes were identified in PPI network, and the early growth response 2 (EGR2) was highlighted. In vitro. We confirmed the EGR2 was upregulated induced by hypoxia and revealed the upregulated EGR2 aggravates pro-inflammation and pro-apoptotic genes expression. In addition, EGR2 knockout mitigates hypoxia-induced inflammation and apoptosis in cardiomyocytes.

**Conclusion:**

The present study identified the EGR2 was a hub gene in myocardial damage during MI process, the excessive EGR2 aggravates hypoxia-induced myocardial damage by accelerating inflammation and apoptosis in vitro. Therefore, targeting EGR2 offers a potential pharmacological strategy for myocardial cell rescue in MI.

**Supplementary Information:**

The online version contains supplementary material available at 10.1186/s12872-022-02814-3.

## Introduction

Myocardial infarction (MI) is one of the most common cardiovascular events worldwide [1–3]. Although advances in interventional cardiology and pharmacological strategies have led to a decline in all-cause mortality from MI over the past few decades, MI remains one of the most common causes of morbidity and mortality all over the world [2, 3]. MI is characterized as myocardial cell ischemia and hypoxia due to coronary artery occlusion, then, the inflammation and cell apoptosis was triggered [4]. The onset of MI, hypoxia leads to cardiomyocytes damage and necrosis, the damaged cardiomyocytes release pro-inflammatory chemokines to recruit inflammatory cells to MI zone and clear necrotic cardiomyocyte [4, 5]. However, the myocardial reperfusion following percutaneous coronary intervention (PCI) exacerbates the pro-inflammatory response and myocardial injury [1].

A larger number of studies have demonstrated the release of damage-associated molecular patterns or DAMPs (such as ATP, mtDNA, RNA, and HMBGB1) induce a pro-inflammatory response following MI, which mediates cardiomyocyte death via cytokines, mitochondrial dysfunction and inflammasome formation [4, 6, 7]. Therefore, it’s necessary to explore the mechanism of inflammation and apoptosis induced by myocardial hypoxia to find new targets for mitigating myocardial injury after MI. Previously studies showed the early growth response gene family (EGR) was play key roles in inflammation and apoptosis in physiology and pathophysiology [8, 9]. In human, the EGR1 and EGR2 were highly expressed in cardiomyocytes, the EGR1 were demonstrated play important roles in myocardial injury via oxidative stress, ferroptosis and apoptosis signaling pathways in MI process [9, 10], but the roles of EGR2 in MI remain unrevealed.

Early growth response 2 (EGR2) is one of the early growth response gene families, contains three cyc2-His2 zinc fingers binding to the same cognate GC-rich consensus DNA binding motif of 28–30 amino acids [11]. EGR2 is expressed in many tissue and different cell types, and plays key roles in response to inflammation, apoptosis and tissue damage [11]. In immune cells, study has demonstrated that EGR2 regulates the inflammatory responses of PD-1 high MP CD4 T cells and maintains their adaptive immune fitness [12]. Meanwhile, the EGR2 expression in T cells is mediated through IFNγ/STAT1 and IL-6/STAT3 signaling pathway [13]. EGR2 has been reported to directly activate the expression of pro-apoptotic proteins of Bcl-2 family, BNIP3L and BAK, which activates caspase families, such as caspase-3, caspase-8 and caspase-9 [14]. In cancer, NFAT2 inhibits the growth of hepatocellular carcinoma by inducing EGR2 expression [15]. In the MI process, the miR-150 plays a cardioprotective role by directly repressing EGR2 expression in cardiomyocytes [16]. A recent study showed that the inhibition of long non-coding RNA MIAT ameliorates myocardial dysfunction induced by myocardial infarction via MIAT/miR-10a-5p/EGR2 axis [17]. Although EGR2 is involved in inflammation and apoptosis, the function of EGR2 in the MI process remains unclear.

In the present study, we analyzed the gene expression in an animal model of MI and attempted to identify the hub genes and potential therapeutic targets. We downloaded the gene expression data for heart tissue of mouse with MI and sham control from Gene Expression Omnibus (GEO) database and screened the differentially expressed genes (DEGs). Gene Ontology (GO) and Kyoto Encyclopedia of Genes and Genomes (KEGG) pathway functional enrichment analysis was performed to underlying the pathway changes of MI compared with sham controls. Protein–protein interaction (PPI) network analysis was used to find the potential hub-genes in MI, and the EGR2 was highlighted in the top20 Hub-genes. We then validated the expression and revealed the function of EGR2 in hypoxia induced human cardiomyocyte cell line. Our data suggested the EGR2 is a potential therapeutic target for the treatment of myocardial injury in MI.

## Methods

### Data sources

The datasets of GSE71906 was downloaded from the GEO database (http://www.ncbi.nlm.nih.gov/geo/). A total of 12 samples were collected in the GSE71906 dataset, 6 samples from MI zone of mouse heart and 6 from normal sham control hearts, and all the samples were obtained post 8 h of surgery. GPL8321 platform was used for GSE71906 sequencing ([Mouse430A_2] Affymetrix Mouse Genome 430A 2.0 Array).

### Data preprocessing of DEGs

The Uniform manifold approximation and projection (UMAP) plot and volcano plot were obtained by GEO2R (https://www.ncbi.nlm.nih.gov/geo/geo2r). The dataset of GSE71906 was download with the CEL format, and the gene expression profiling analysis were preprocessed using Robust Multichip Average algorithm in the “affy” and “affyPLM” packages within Bioconductor (http://www.bioconductor.org) by a R × 64 4.1.1 software (R Foundation for Statistical Computing, Vienna, Austria). After correcting for background, and performing quantile normalization, the DEGs analysis was performed with the 13,015 genes. 235 genes were met the adjusted p value < 0.05 and logFC > 1 (upregulated genes) or logFC < −1 (downregulated genes), and were considered as DEGs for the subsequent analysis, the unqualified genes were discarded.

### Heatmap analysis

Heatmap analysis were performed with the DEGs of MI and sham control samples by means of ImageGP (http://www.ehbio.com/ImageGP/index.php/Home/Index/ index.html).

### GO analysis and KEGG pathway enrichment analysis

Gene Ontology (GO) analysis is a functional analysis associating DEGs with GO categories, and involve cell composition (CC), biological process (BP)and molecular function (MF). Kyoto Encyclopedia of Genes and Genomes (KEGG) pathway analysis offers biological pathways of DEGs genes related to diseases. The DEGs were used for GO and KEEG analysis by an online biological function database, Database for Annotation, Visualization and Integrated Discovery (DAVID, http://david.ncifcrf.gov), The enrichment index with p < 0.05 was considered to be statistically significant. The enrichment plots were constructed by ImageGP.

### Protein–protein interaction (PPI) network construction and hub genes identification

To explore possible protein–protein interaction network interactions, the DEGs were mapped to the Search Tool for the Retrieval of Interacting Genes/ Proteins database (STRING, https://string-db.org/). The PPI pairs were extracted with a minimum required interaction score > 0.4. The interactions were download from STRING and used to construct a PPI network by Cytoscape software platform (3.9.0). The nodes with higher connectivity degree were considered important in maintaining the stability of the PPI network, therefore, the CytoHubba plugin for Cytoscape software was used to indentify the top20 hub genes.

### Cell culture

The human cardiomyocyte cell line AC16 and HEK293T cell line were purchased from the American Type Culture Collection (ATCC). Cells were cultured in DMEM medium (Invitrogen, Carlsbad, USA) with 10% FBS (Invitrogen) under 5% CO2 at 37 °C. For hypoxia treatment, the AC16 cells were maintained in a hypoxic incubator (N2 94%, O2 1% and CO2 5%) for 12 h in medium deprived of serum and glucose.

### Stable cell line construction

The full-length sequence of human EGR2 consensus CDS region was cloned and insert to pHAGE vector which containing a Flag tag to construct the EGR2 expression vector pHAGE-Flag-EGR2. The pHAGE-Flag-EGR2 or the empty pHAGE-Flag vector was co-transfected into HEK293T with lentivirus packaging plasmids, psPA × 2 (12260, Addgene) and pMD2.G (12259, Addgene) to obtain the lentivirus. Then, the lentivirus was used to infect AC16 cells with polybrene (10 μg/mL). 48 h later, the EGR2 stable overexpressed cell line were screened by 2 μg/mL puromycin (A1113803; Gibco, USA), and the expression was verified by Western blot analysis. Primers: Forward 5′-TCGGGTTTAAACGGATCCATGGCATGATCAACATTGAC-3′, Reverse 5′-GGGCCCTCTAGACTCGAGAGGTGTCCGGGTCCGAGAGG-3′.

### EGR2-knockout cell line generation

The EGR2-Knockout cardiomyocyte were generated using a pX459 vector (62988, Addgene). The human EGR2-sgRNA was synthesized and cloned into pX459, then, the recombinated vector was transfected into AC16 cells. Homozygous EGR2-knockout cells were generated from a monoclonal line after puromycin selection. The target sequence for human EGR2 sequence was 5′-CAATCCGTAACTTTACCCTG-3′.

### Reverse transcription-quantitative polymerase chain reaction (RT-qPCR)

Total RNA from AC16 was extracted using TRIzol reagent (Invitrogen, CA, USA) according to the manufacturer’s protocol. RNA was converted to cDNA using the HiScript II Q RT SuperMix for qPCR (+ gDNA wiper) (R223-01, Vazyme, China). qPCR was conducted using ChamQ SYBR qPCR Master Mix (#Q311-02, Vazyme, China). GAPDH were used as endogenous control, all samples were run in triplicate. The data analyses were performed with SDS 2.2.2 software. Primers: EGR2, Forward 5′-TCAACATTGACATGACTGGAGAG-3′, Reverse 5′-AGTGAAGGTCTGGTTTCTAGGT-3′; GAPDH, Forward 5′-GGAGCGAGATCCCTCCAAAAT-3′, Reverse 5′-GGCTGTTGTCATACTTCTCATGG-3′; IL-6, Forward 5′-ACTCACCTCTTCAGAACGAATTG-3′, Reverse 5′-CCATCTTTGGAAGGTTCAGGTTG-3′; IL-1b, Forward 5′-ATGATGGCTTATTACAGTGGCAA-3′, Reverse 5′-GTCGGAGATTCGTAGCTGGA-3′; CCL2, Forward 5′-CAGCCAGATGCAATCAATGCC-3′, Reverse 5′-TGGAATCCTGAACCCACTTCT-3′; TNF, Forward 5′-CCTCTCTCTAATCAGCCCTCTG-3′, Reverse 5′-GAGGACCTGGGAGTAGATGAG-3′; BAX, Forward 5′-CCCGAGAGGTCTTTTTCCGAG-3′, Reverse 5′-CCAGCCCATGATGGTTCTGAT-3′; BAD, Forward 5′-CCCAGAGTTTGAGCCGAGTG-3′, Reverse 5′-CCCATCCCTTCGTCGTCCT-3′; BCL2, Forward 5′-GGTGGGGTCATGTGTGTGG-3′, Reverse 5′-CGGTTCAGGTACTCAGTCATCC-3′.

### Western blot assay

Total protein was extracted by RIPA lysis buffer (#P0013C, Beyotime Institute of Biotechnology, China) and quantified using a Pierce BCA Protein Assay Kit (#P0012S, Beyotime Institute of Biotechnology, China). The total protein was electrophoresed by 10% SDS-PAGE and transferred to PVDF membranes (#AR0136-04, BOSTER, China). The membranes were blocked in non-fat dry milk (~ 5%) for 60 min at room temperature and incubated with special primary antibodies at 4 °C overnight. After washing, the membranes were incubated with peroxidase-conjugated secondary antibody (anti-rabbit) at room temperature for 60 min. Protein expression levels were visualized using enhanced chemiluminescence kit (#P0020; Beyotime Institute of Biotechnology, China). Images were visualized with a ChemiDoc MP Imaging System (Bio-Rad, Hercules). Primary antibodies: EGR2 antibody, (#ab245228, dilution 1:1000, Abcam, UK); GAPDH antibody (#A19056, dilution 1:1000, ABclonal, China).

### Statistical analysis

All data were presented as mean ± standard deviation (SD) of three independent experiments with the software GraphPad Prism 7. Significance was assessed by one-way ANOVA followed by Tukey’s test. All the statistics were performed in SPSS 22.0 (SPSS Inc., Chicago, Illinois, USA). A p value < 0.05 was considered statistically significant.

## Results

### Bioinformation analysis from the GSE71906 dataset and identification of DEGs

**Fig. 1:**
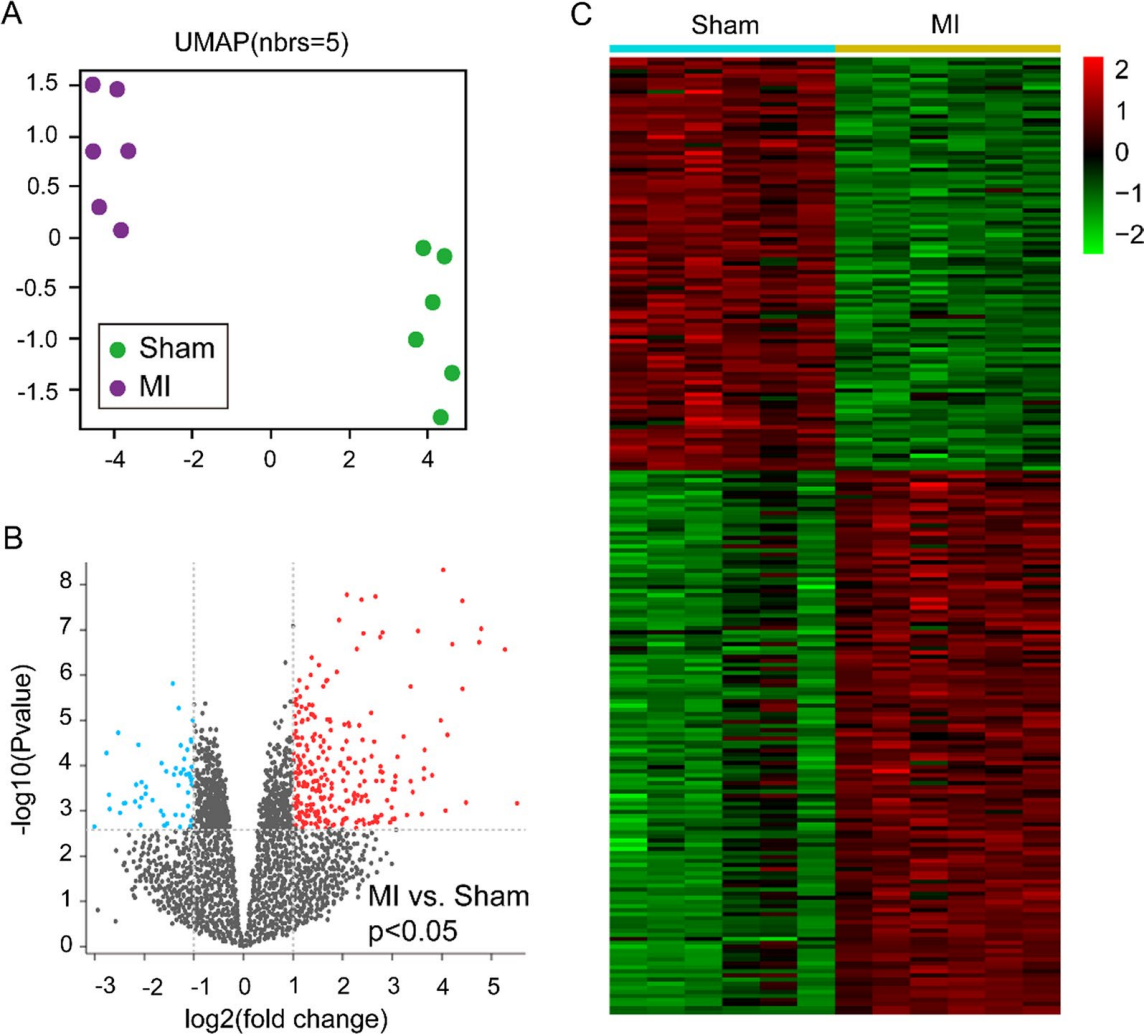
Differentially expressed genes in MI compared to sham controls. **A** UMAP plot was used to visualize the relationships between samples, purple dots represent the MI samples and the green dots represent the sham control samples. **B** Volcano plot of p values as a function of the weighted fold change for genes in MI and sham control samples. gray dots represent genes that are not significantly differentially expressed (log(fold change) < −1 or > 1, p > 0.05), red dots represented the gens that are significantly upregulated in MI situation (log(fold change) > 1, p < 0.05), the baby blue dots represent the genes that are significantly downregulated in MI samples (log(fold change) < −1, p < 0.05). **C** Heatmap shows the expression profiles for the 235 differentially expressed genes (102 downregulated and 133 upregulated). The red to green color gradient indicates a high to low level of expression. For **A** to **C**, n = 6 samples in each group

To investigate the expression of transcriptome in MI heart tissue, we sighted and downloaded the GSE71906 dataset from GEO DataSets. The UMAP analysis showed that 6 samples were clustered into the MI group and another 6 samples were clustered into the sham control group (Fig. 1A), indicating the genomic expression in MI was significant difference compared to sham controls. After data processing, a total of 13,015 genes were recognized from GSE71906 dataset, and 235 genes were considered as DEGs which met the adjusted p value < 0.05 and logFC > 1 (upregulated genes) or logFC < -1 (downregulated genes) (Fig. 1B). Additionally, the heatmap analysis exhibited the 102 downregulated and 133 upregulated genes were contained in the 235 DEGs (Fig. 1C). The top 20 upregulated genes were listed in Table 1 and most of them were related to inflammation and apoptosis, including interleukin 6 (*Il6*), *Fos*, C-X-C motif chemokine ligand 5 (*Cxcl5*), C-X-C motif chemokine ligand 1 (*Cxcl1*), C-X-C motif chemokine ligand 5 (*Cxcl2*) and early growth response 1 (*Egr1*).

**Table 1:** The top 20 upregulated and downregulated genes of DEGs

Gene symbol	logFC	p Value	Gene symbol	logFC	p Value
Hspa1a	5.382	0.000	Cdh11	− 2.234	0.000
Hspa1b	4.833	0.000	Fign	− 1.571	0.000
Il6	4.547	0.000	Fgf16	− 1.564	0.000
Ptx3	4.335	0.000	Rsrp1	− 1.524	0.000
Atf3	4.078	0.000	Cth	− 1.496	0.001
Fos	3.855	0.000	Npr3	− 1.469	0.000
Ch25h	3.286	0.000	Taf1b	− 1.451	0.000
Egr1	3.196	0.000	Ttc8	− 1.428	0.000
Cyr61	3.114	0.000	Inmt	− 1.422	0.002
Cxcl5	2.952	0.007	Upk3b	− 1.408	0.012
Fosb	2.944	0.000	Rad1	− 1.395	0.000
Socs3	2.778	0.000	Gstt3	− 1.381	0.000
Cxcl1	2.664	0.001	Tril	− 1.345	0.000
Il1r2	2.645	0.000	Mis12	− 1.344	0.000
Sphk1	2.620	0.000	Zfp958	− 1.331	0.000
Cxcl2	2.614	0.000	Hist3h2a	− 1.328	0.000
Mt2	2.570	0.003	Trmt1l	− 1.327	0.000
Thbs1	2.547	0.000	Cd48	− 1.316	0.000
Nppb	2.514	0.003	Aspa	− 1.272	0.000
Adamts1	2.468	0.000	Ubxn2b	− 1.262	0.000

### Functional enrichment analyses of DEGs

**Fig. 2:**
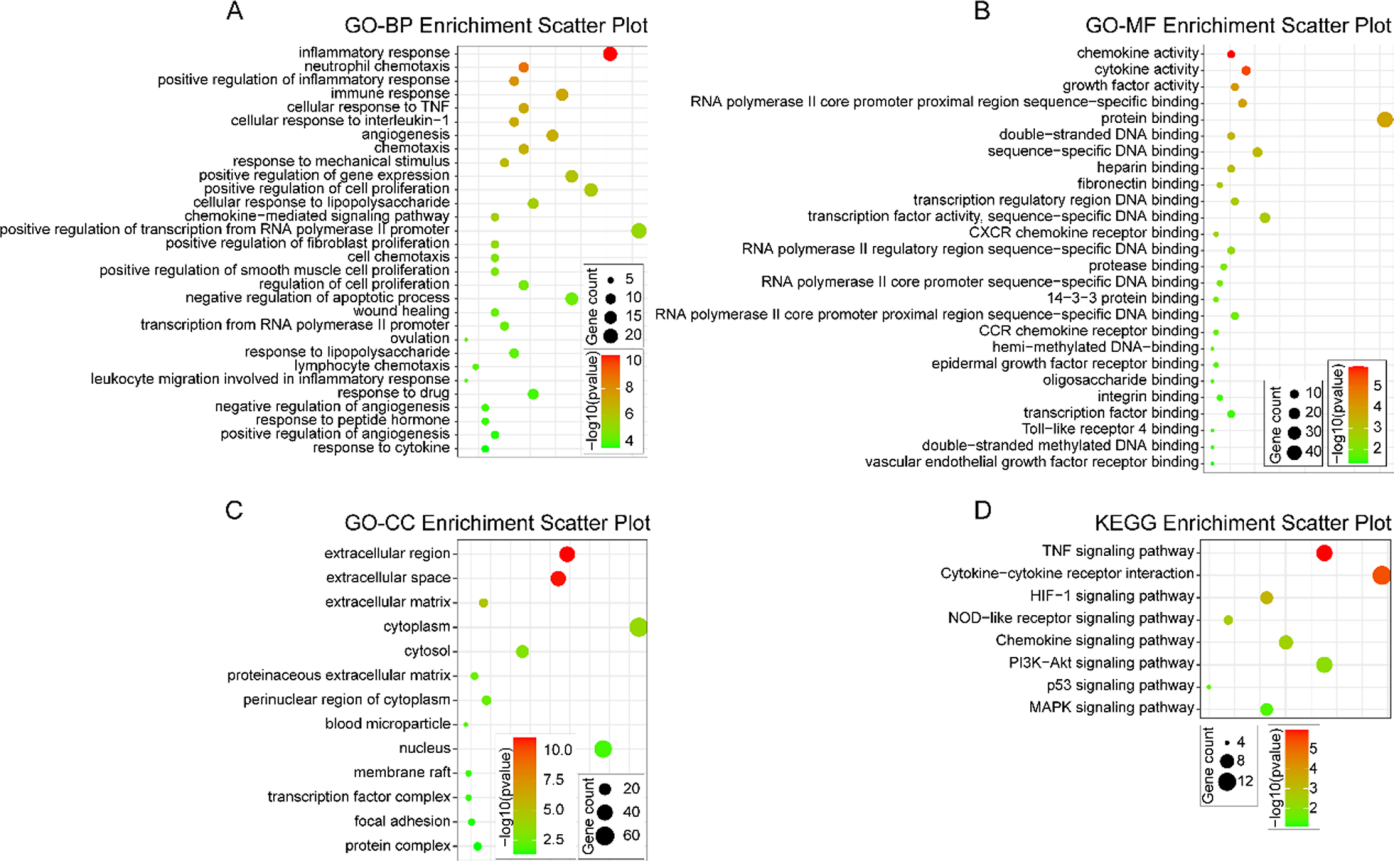
Gene ontology (GO) and Kyoto Encyclopedia of Genes and Genome (KEGG) enrichment analysis. **A** The top30 significant GO biological process terms; **B** the most significant GO molecular function terms; **C** The significant GO cellular component terms; **D** The enriched KEGG pathway terms. p < 0.05 for all significant GO terms and KEGG pathway terms

In order to further understand the functions of the EDGs in MI process, the comprehensive analysis, GO and KEGG, of the functions of these DEGs was performed (Fig. 2). In the GO enrichment analysis, 164 GO terms were enriched (p < 0.05) including 125 biological process (BP) terms, 13 cellular component (CC) terms and 26 molecular function (MF) terms. As to BP, the upregulated DEGs were mostly enriched in inflammation related and apoptotic terms (Fig. 2A), including inflammatory response, neutrophil chemotaxis, positive regulation of inflammatory response, immune response, cellular response to TNF, cellular response to interleukin-1, chemokine-mediated signaling pathway, leukocyte migration involved in inflammatory response, response to cytokine wound healing, negative regulation of apoptotic process. For MF, the upregulated DEGs were mostly enriched in chemokine activity, cytokine activity, growth factor activity, protein and DNA binding, CXCR chemokine receptor binding, CCR chemokine receptor binding, Toll-like receptor 4 binding terms (Fig. 2B). In CC, the extracellular region, extracellular space, extracellular matrix, cytoplasm, focal adhesion terms were enriched (Fig. 2C). For the KEGG pathway enrichment analysis, the upregulated DEGs were most significantly enriched in 8 pathways which all related with hypoxia, inflammation and cell apoptosis (Fig. 2D), including TNF signaling pathway, Cytokine-cytokine receptor interaction, HIF-1 signaling pathway, p53 signaling pathway and MAPK signaling pathway. The details are shown in Table 2.

**Table 2:** The KEGG pathway enrichment terms of the upregulated DEGs in MI

KEGG term	Pathway name	Gene number	p value
mmu04668	TNF signaling pathway	10	1.20E − 06
mmu04060	Cytokine-cytokine receptor interaction	13	4.29E − 06
mmu04066	HIF-1 signaling pathway	7	5.20E − 04
mmu04621	NOD-like receptor signaling pathway	5	0.002455
mmu04062	Chemokine signaling pathway	8	0.003342
mmu04151	PI3K-Akt signaling pathway	10	0.008064
mmu04115	p53 signaling pathway	4	0.029251
mmu04010	MAPK signaling pathway	7	0.039293

### Protein–protein interaction network construction and hub gene identification

**Fig. 3:**
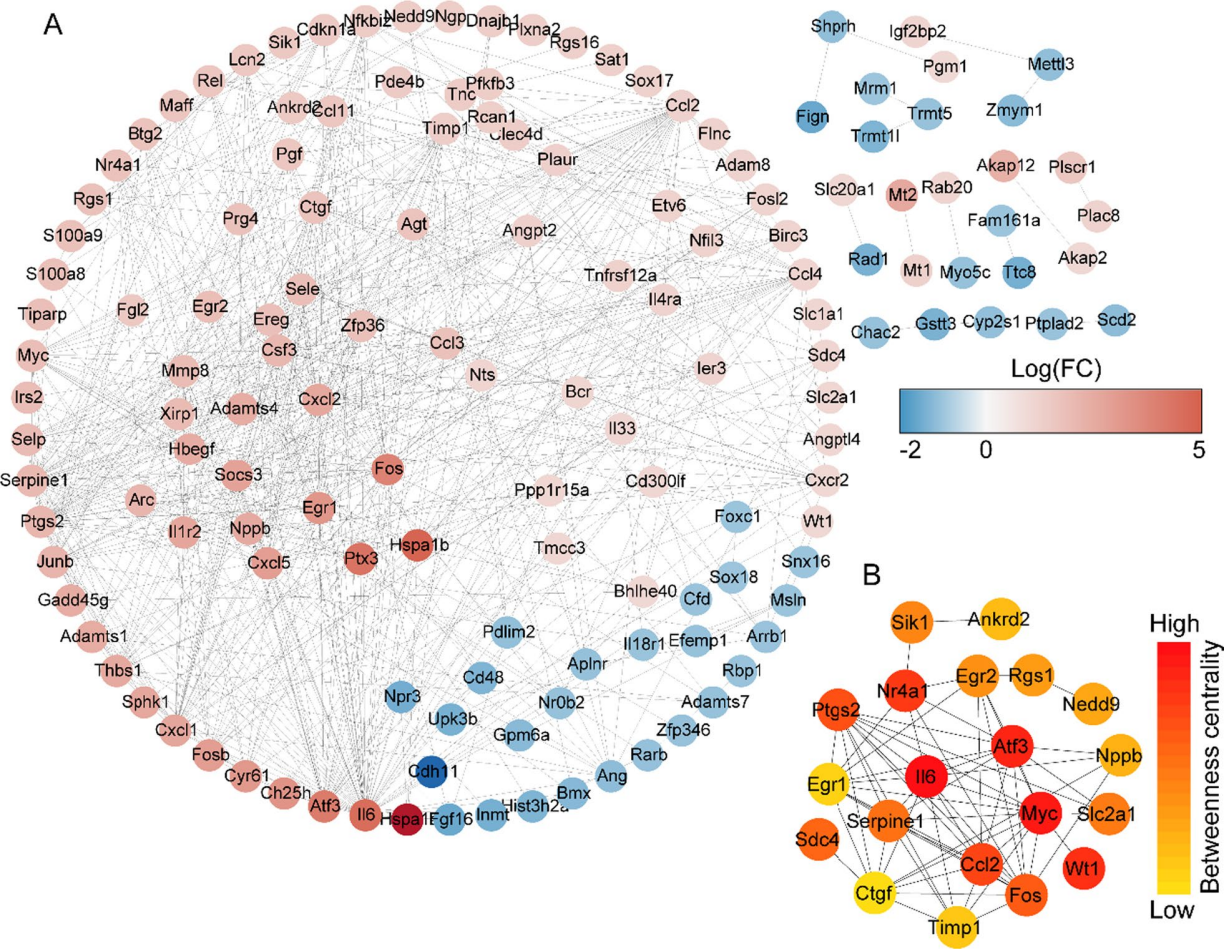
Protein–protein interaction (PPI) networks and hub genes of differential expressed genes in MI samples compared with sham controls. **A** PPI networks show the interaction of DEGs in MI samples compared with sham controls. The red to blue color gradient indicates an upregulation to downregulation of DEGs indicated by logFC. **B** The top 20 hub genes from DEGs in MI samples compared with sham controls. The red to yellow color gradient indicates a high to low centrality

We subsequently constructed a PPI network and evaluated the interaction through the STRING database to explore the association between 235 DEGs. After the calculation of STRING database, the 146 nodes and 621 connections were generated, and the PPI was visualized by Cytoscape software (Fig. 3A). Further, the top 20 DEGs with high degree of connectivity were selected as the hub genes in MI (Fig. 3B). Most of the hub genes were related to inflammation and apoptosis (Table 3), and were reported to play roles in MI, such as Il6, C–C motif chemokine ligand 2 (*Ccl2*), Fos, activating transcription factor 3 (*Atf3*), WT1 transcription factor (*Wt1*), and *Erg1*. However, the function of hub gene *Egr2* in MI remains unclear.

**Table 3:** The hub genes of DEGs in MI and sham control samples

Gene symbol	logFC	pValue	Connectivity score	Gene symbol	logFC	pValue	Connectivity score
Il6	4.547	0.000	3574.6	Slc2a1	1.079	0.002	700.0
Myc	1.852	0.000	2970.6	Sik1	1.528	0.000	696.0
Atf3	4.078	0.000	1385.0	Egr2	1.875	0.005	555.9
Wt1	1.029	0.000	1372.0	Rgs1	1.757	0.000	539.0
Nr4a1	1.724	0.000	972.1	Nedd9	1.405	0.000	531.3
Ccl2	1.216	0.004	906.2	Nppb	2.514	0.003	472.8
Ptgs2	2.128	0.000	852.2	Ankrd2	1.509	0.000	468.0
Fos	3.855	0.000	847.7	Timp1	1.350	0.010	426.2
Sdc4	1.082	0.001	730.1	Egr1	3.196	0.000	424.9
Serpine1	2.026	0.002	712.6	Ctgf	1.713	0.000	422.3

### EGR2 overexpression deteriorates the hypoxia induced inflammation and apoptosis in cardiomyocytes

**Fig. 4:**
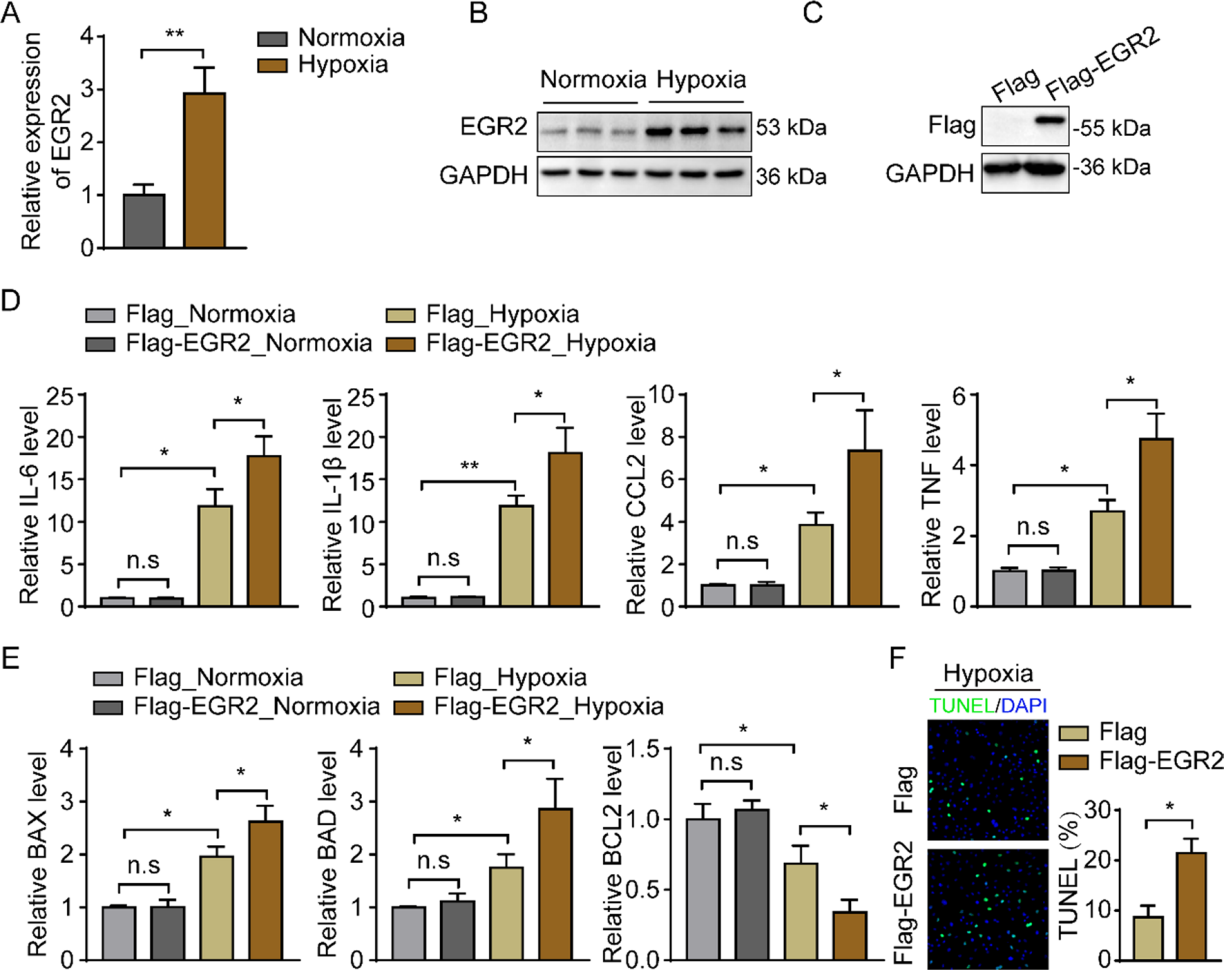
EGR2 overexpression deteriorates the hypoxia induced inflammation and apoptosis in cardiomyocytes. **A** The relative EGR2 mRNA expression level in cardiomyocytes induced by hypoxia indicated by qPCR, GAPDH was used as internal control, n = 3 in each group; **B** Western blot analysis of the relative EGR2 protein expression level in cardiomyocytes induced by hypoxia, GAPDH was served as internal control, n = 3 in each group; **C** Western blot analysis of EGR2 protein expression in cardiomyocytes with EGR2 gene overexpression mediated by lentiviral vector. GAPDH was served as internal contro; **D** the inflammation related genes expression in hypoxia-induced cardiomyocytes compared to normoxia-cultured cells detected by qPCR assays. GAPDH was served as internal control, n = 3 in each group; **E** qPCR analysis of the genes expression related to apoptosis in normoxia-cultured and hypoxia-induced cardiomyocytes. GAPDH was used as internal control, n = 3 in each group. **F** TUNEL staining show the apoptotic cells in hypoxia-induced cardiomyocytes, n = 3 in each group. *p < 0.05, **p < 0.01, n.s., no significant; All data are shown as the mean ± SD. Abbreviations: *IL-6* interleukin 6, *IL-1b* interleukin 1b, *CCL2* C–C motif chemokine ligand 2, *TNF* tumor necrosis factor, *BAX* BCL2 associated X, *BAD* BCL2 associated agonist of cell death, *BCL2* B cell lymphoma protein-2

To investigate the function of EGR2 in MI, we first detected the expression of EGR2 in hypoxia-induced human cardiomyocyte cell line in vitro. The EGR2 mRNA and protein relative expression level were upregulated which indicated by qPCR and Western blot assays (Fig. 4A, B), and this result was consistently to the DEGs in MI samples. Next, we generated an EGR2 stable overexpressed cardiomyocyte cell line based on AC16 cells by lentivirus. The Western blot analysis showed the overexpressed cell line was successfully constructed (Fig. 4C). Since the major gene expression changes in myocardial tissue during myocardial infarction process are the activation of inflammatory and apoptotic signaling pathways, we examined the expression of inflammatory and apoptotic genes in hypoxia-induced cardiomyocytes in vitro to evaluate the role of EGR2 overexpression in MI. The data showed the EGR2 overexpression remarkably increases the pro-inflammatory genes (IL-6, IL-1b, CCL2 and TNF) expression in cardiomyocytes under hypoxia condition (Fig. 4D), as well as the apoptotic genes (BAX and BAD), meanwhile, the anti-apoptotic gene, BCL2, was inhibited (Fig. 4E). Additionally, the TUNEL staining showed the apoptotic cells were remarkably increased in EGR2 overexpressed cardiomyocytes compared to Flag control (Fig. 4F). Taken together, this part suggested that the EGR2 in cardiomyocytes was upregulated induced by hypoxia in vivo and in vitro, and the excessive expressed EGR2 exacerbates inflammatory response and apoptosis in hypoxia-induced cardiomyocytes.

### EGR2 deficiency mitigates the hypoxia-induced inflammatory response and apoptosis in cardiomyocytes

**Fig. 5:**
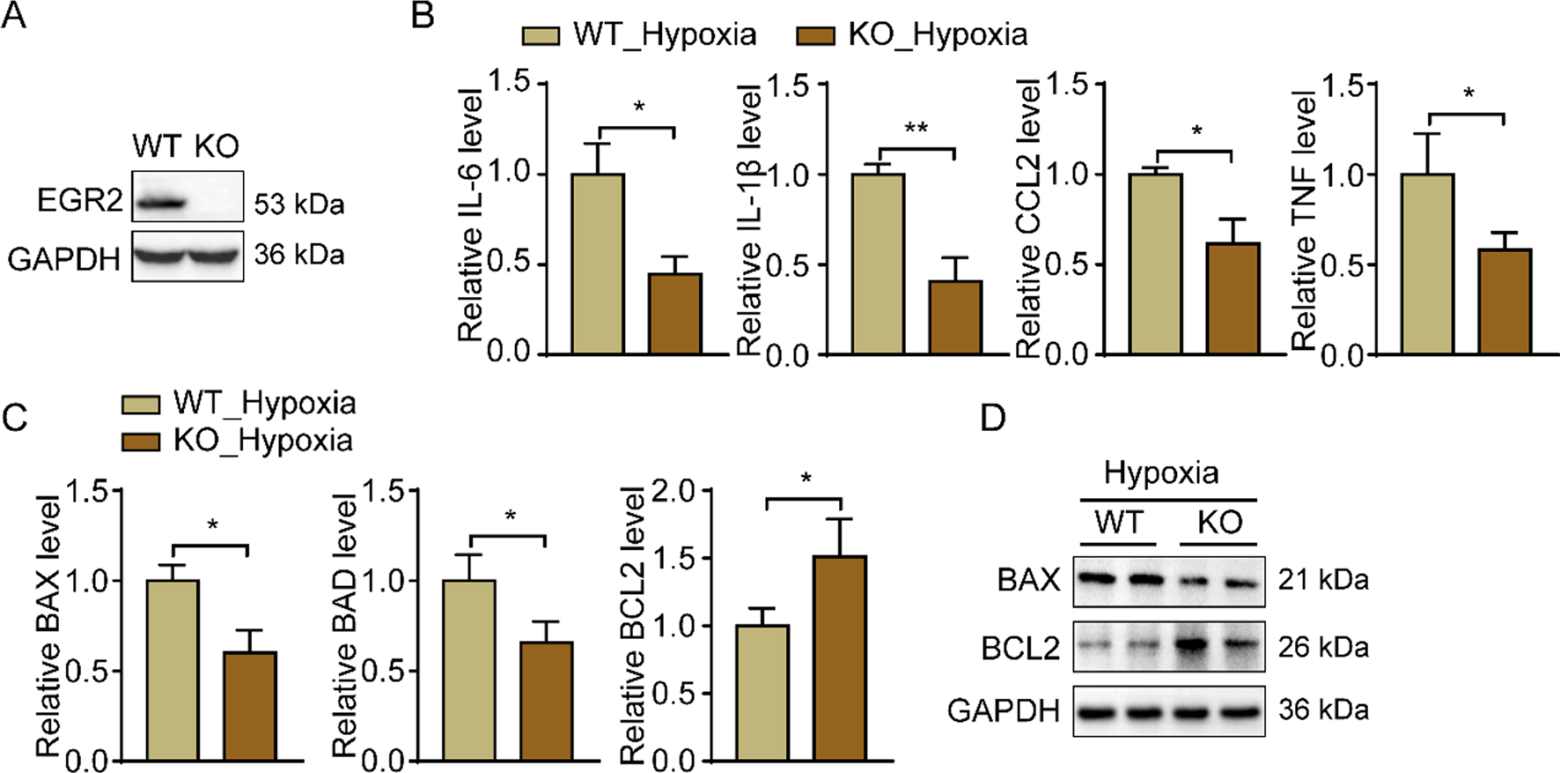
EGR2 deficiency mitigates the hypoxia-induced inflammatory response and apoptosis in cardiomyocytes. **A** Western blot analysis of the relative EGR2 protein expression level in WT and EGR2 knockout cardiomyocytes, GAPDH was served as internal control; **B** the inflammation related genes expression in hypoxia-induced WT and EGR2-KO cardiomyocytes detected by qPCR assays. GAPDH was served as internal control, n = 3 in each group; **C** qPCR analysis of the genes expression related to apoptosis in hypoxia-induced WT and EGR2-KO cardiomyocytes. GAPDH was used as internal control, n = 3 in each group; **D** representative Western blot images of BAX and BCL2 expression in hypoxia-induced WT and EGR2-KO cardiomyocytes. GAPDH was used as internal control, n = 3 in each group. *p < 0.05, **p < 0.01; All data are shown as the mean ± SD. Abbreviations: *WT* wild type, *KO* knockout, *IL-6* interleukin 6, *IL-1b* interleukin 1b, *CCL2* C–C motif chemokine ligand 2, *TNF* tumor necrosis factor, *BAX* BCL2 associated X, *BAD* BCL2 associated agonist of cell death, *BCL2* B cell lymphoma protein-2

We then explored whether EGR2 lacking mitigates the hypoxia-induced inflammatory response and apoptosis in cardiomyocytes. We generated an EGR2 knockdown cardiomyocytes cell line by transduction with a lentiviral vector which contains a sgRNA target to EGR2, the Western Blots analysis demonstrated the EGR2-KO cells were successfully constructed (Fig. 5A). After hypoxia treatment, the qPCR and Western blot analysis showed the EGR2 deficiency significantly reduces the pro-inflammatory (IL-6, IL-1b, CCL2 and TNF) and apoptotic genes (BAX and BAD) expression in cardiomyocytes compared to the WT cells (Fig. 5B–D). Meanwhile, the anti-apoptotic gene, BCL2, was upregulated in EGR2-KO cells (Fig. 5C, D). Those results indicated that lack of EGR2 is beneficial to decreasing the inflammatory response and apoptosis in hypoxia-induced cardiomyocytes.

## Discussion

MI is a common cause of mortality worldwide because of the irreversible damage to the non-renewable cardiomyocytes, and aggravates cardiac inflammation and cell apoptosis [18]. However, the pathogenesis of MI remains poorly understood. To explore the changes of gene expression and identify the hub genes in MI, we screened and analyzed a microarray dataset from MI mice heart tissue.

Compared with the previous studies, our investigation provides new insights into the pathogenesis of MI. *Bennardo M *et al. produced the GSE71906 dataset, the authors established the mice MI models within a 2-h time window either shortly after lights on or lights off, respectively, to observe the early remodeling response at 8 h after infarction [19]. They found that the day-night dependence of gene expression and inflammatory responses in the remodeling murine heart post-myocardial infarction [19]. In the present study, we explored global genetic changes and attempted to figure out the hub genes in mice after MI. We first identified the 235 significant DEGs between MI and sham control samples. Most of the top 20 upregulated genes were reported related to inflammation and apoptosis. For instance, IL6 is often used as an evaluative indicator for inflammatory response [20] and there have been many studies of it related to MI [21]. FOS is a subunit of activator protein-1 (AP-1) and plays an important role in inflammation and cell apoptosis [22–24]. In addition, the AP-1 significantly increased in IM tissues [25, 26] and involved in the process of cardiac injury after MI [27]. We then conducted GO and KEGG pathway enrichment using the upregulated DEGs. As expected, the analysis showed that the pathways related to inflammation and cell apoptosis.

The PPI and hub-gene analysis exhibited the key genes in the MI process, and two members of the EGR family were highlighted. Many previous studies have revealed the function of EGR1 in the MI process. *Bhindi R *et al*.* found EGR1 is a key contributor to myocardial ischemia reperfusion injury [28], and targeting EGR1 by DNA-zymes reduced the infarct size following myocardial ischemia reperfusion [29]. Some scholars have pointed out that EGR1 is a key player in myocardial cell injury in MI process [9], and it was supported by later research [10, 30–32]. Although *Tang Y* and *Cao X* revealed the miRNA150/10a-5p-EGR2 axis play roles in MI [16, 17], the function of EGR2 in MI remains unclear.

Previous study suggested EGR2 was a pro-apoptotic gene. *Unoki M* and *Nakamura Y* found that EGR2 induces apoptosis in a large proportion of these lines by altering the permeability of mitochondrial membranes, releasing cytochrome c and activating caspase-3/8/9 by directly transactivates expression of BNIP3L and BAK [14]. In tumor, study have showed EGR2 knockdown promotes gastric cancer cell growth and inhibited their apoptosis [33], *Wang J *et al*.* revealed the EGR2 mediated the function of NFAT2 in inhibition of the invasion and malignancy of hepatocellular carcinoma [15]. *Zeng T *et al*.* found the EGR2 was upregulated through lncRNA-AF113014-miR-20a axis and inhibits the proliferation of hepatocellular carcinoma cells [34]. In Hirschsprung's disease, EGR2 may mediated the downregulated miR-140-5p to promote apoptosis in SH-SY5Y cells [35]. In the present study, we generated the EGR2 overexpression and knockout cell lines based on AC16 cells and established the MI cell model in vitro. Our data showed the EGR2 was upregulated in myocarcytes induced by hypoxia, the excessively overexpressed EGR2 facilitates the hypoxia-induced pro-inflammatory and pro-apoptotic genes expression in myocarcytes. Meanwhile, these phenotypes were reversed in EGR2 knockout myocarcytes. Therefore, these data indicated the hub gene, EGR2, deteriorates cardiac injury by aggravating inflammation and cell apoptosis in the MI process. Thus, our study identified the role of EGR2 in myocardial apoptosis.

Inflammation post-MI has been the focus of cardiovascular research as it influences the remodeling process of the ischemic heart, which critically determines the clinical outcome of MI patients [36]. As we know, inflammation is critical for initiation of the natural wound healing process, in the MI, an appropriate inflammatory response helps scar formation of heart tissue and recovery of cardiac function. However, it is commonly known that inflammation is a double-edged sword. Several studies have shown that high levels of inflammation post-MI leads to an increase of scar volume and lead to poor ventricular remodeling and deterioration of cardiac function [36, 37]. Thus, controlling the intensity of the inflammatory response after MI is particularly important for the recovery of cardiac function. *Hausenloy *et al. showed a multi-targeted approach and combining anti-inflammatory agents offering a better approach to reducing MI size in STEMI patients [38]. *Arslan *et al. revealed anti-inflammation by TLR2 antibodies reduces MI size in both small and large animal AMI models [39, 40]. What’s more, studies showed that genetic and pharmacological inhibition of the NLRP3 inflammasome may reduce MI size and prevent adverse LV remodeling [41, 42]. In the present study, EGR2 knockout exhibited an anti-inflammation role in hypoxia-induced cardiomyocytes, thus, the EGR2 may offer a new potential target for controlling the inflammatory response post-MI. However, the effect of EGR2 on scar formation and myocardial fibrosis after MI should be focused in the future.

## Conclusion

In conclusion, our present study was based on the gene expression dataset obtained from the GEO database, identified 20 hub genes in DEGs between infarcted myocardium and sham controls and highlight the EGR2. We revealed the EGR2 aggravates hypoxia-induced myocardial damage by accelerating inflammation and apoptosis. Therefore, targeting EGR2 offers a potential pharmacological strategy for myocardial cell rescue in MI.

## Supplementary Information

**Additional file 1.** The uncropped gels for Western blots in this study.

## Data Availability

The datasets used and/or analyzed during the current study are available from the GEO dataset. (Data link: https://www.ncbi.nlm.nih.gov/geo/query/acc.cgi?acc=GSE71906).
