# Supplementary figures and images for: EGR2 is a hub-gene in myocardial infarction and aggravates inflammation and apoptosis in hypoxia-induced cardiomyocytes
Source: BMC Cardiovasc Disord. 2022 Aug 15;22:373. doi: 10.1186/s12872-022-02814-3 (PMC9377070; doi:10.1186/s12872-022-02814-3)

# Uncropped gels for Western blots

4B

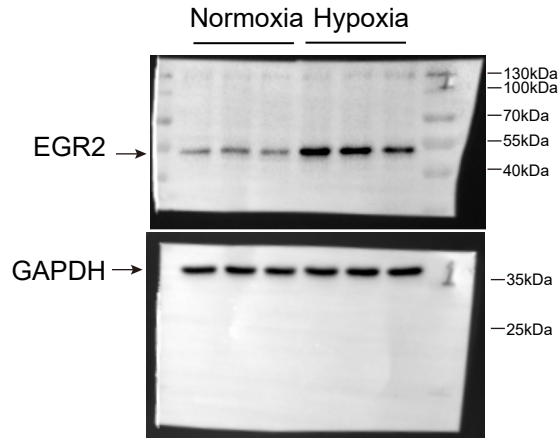

4C

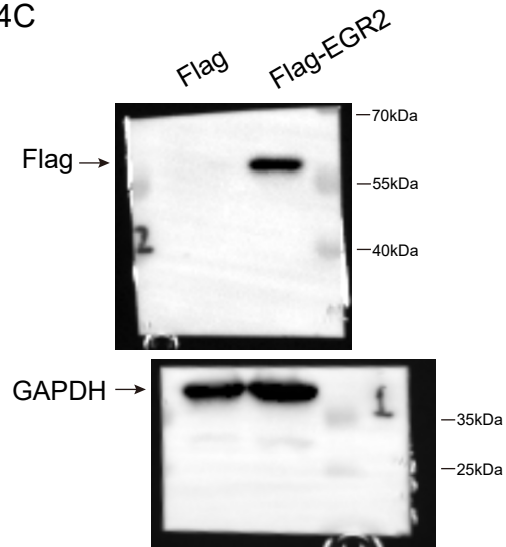

5A

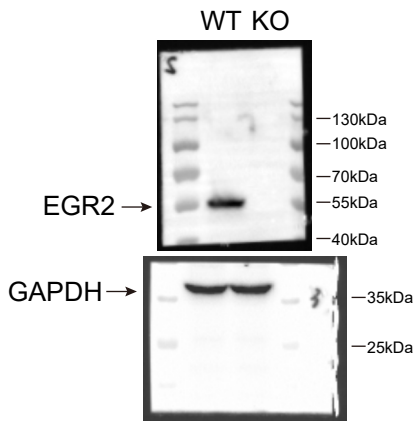

5D

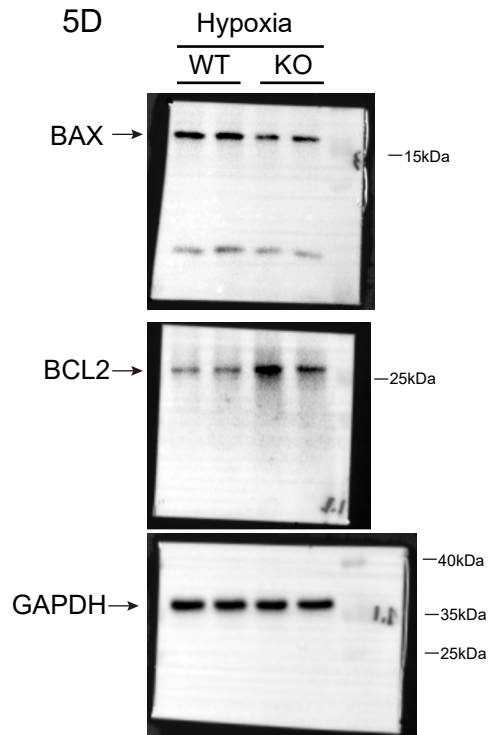

Supplement: Supplementary file 1 — Additional file 1. The uncropped gels for Western blots in this study. [file 12872_2022_2814_MOESM1_ESM.pdf]
